# Hyaluronic Acid-functionalized Hesperidin-loaded Solid Lipid Nanoparticles for Mitigating Oxidative Stress: A Potential Strategy for Radiation-induced Skin Injury

**DOI:** 10.1007/s12010-026-05714-w

**Published:** 2026-04-28

**Authors:** Nileshsinh Chauhan, Anshu Kumar, Anupam Jyoti, Pranav Shah, Nimeet Desai, Manisha Lalan

**Affiliations:** 1 Department of Pharmaceutics, Parul Institute of Pharmacy and Research, Parul University, Waghodia, Vadodara, 391 760 Gujarat India; 2 Department of Life Science, Parul Institute of Applied Sciences, Faculty of Applied Sciences, Parul University, Waghodia, Vadodara, 391 760 Gujarat India; 3 Department of Pharmaceutics, Maliba Pharmacy College, Uka Tarsadia University, Tarsadi, 394 350 Gujarat India; 4 Institute of Life Course and Medical Sciences, University of Liverpool, Liverpool, L7 8TX UK

**Keywords:** Solid lipid nanoparticles, Topical nanocarriers, Topical drug delivery, Hesperidin, Nanoparticle delivery

## Abstract

Radiation-induced skin injury is a common, dose-limiting toxicity of cancer radiotherapy caused by excessive reactive oxygen species (ROS), inflammation, and impaired epidermal repair. Current topical treatments mainly offer symptomatic relief and do not effectively address intracellular oxidative stress. This study reports a hyaluronic acid–functionalized, hesperidin-loaded solid lipid nanoparticle (HSP-HA-SLN) system designed to enhance dermal delivery, skin retention, and intracellular antioxidant activity. HSP-HA-SLNs were prepared by solvent injection and optimized using a Central Composite Design to achieve sub-300 nm particle size and high entrapment efficiency, followed by hyaluronic acid surface functionalization. The optimized formulation was characterized by dynamic light scattering, electron microscopy, thermal, and crystallographic analyses. In vitro drug release, cytocompatibility, intracellular antioxidant activity, and ex vivo skin permeation and retention were evaluated. The nanoparticles exhibited sustained diffusion-controlled drug release, high cytocompatibility, and significantly improved intracellular ROS scavenging compared to free hesperidin. The observed intracellular ROS reduction supports the potential of this system in radiation-induced oxidative stress conditions. Ex vivo studies showed enhanced dermal deposition with minimal surface residue, indicating localized drug reservoir formation. Overall, the HSP-HA-SLN platform overcomes key solubility, permeability, and retention limitations of hesperidin and represents a promising topical nanotherapeutic approach for potential application in radiation-induced skin injury.

**Graphical Abstract:**

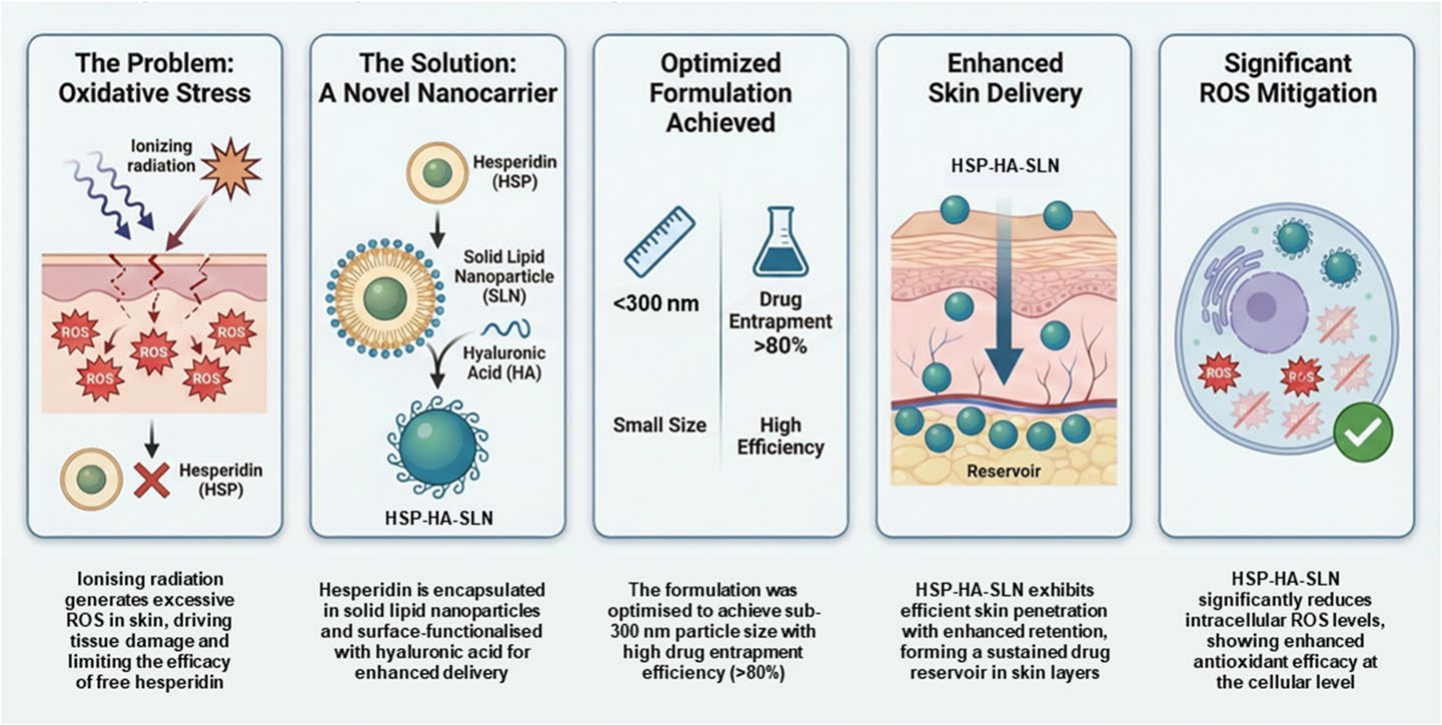

**Supplementary Information:**

The online version contains supplementary material available at 10.1007/s12010-026-05714-w.

## Introduction

One of the most prevalent and clinically difficult complications of radiation therapy is radiation-induced skin injury. Acute skin reactions, which can include erythema, moist desquamation, and, in extreme situations, necrosis, affect nearly 90% of patients [1, 2]. In addition to having a detrimental effect on patient comfort and quality of life, these cutaneous side effects frequently impede treatment compliance, resulting in dose reduction or discontinuation [3]. Direct damage to the DNA, coupled with oxidative stress brought on by ionizing radiation, is biologically responsible for radiation-induced skin damage. Normal epidermal homeostasis is disturbed by a rapid and excessive production of reactive oxygen species (ROS), mitochondrial dysfunction, keratinocyte senescence, endothelial damage, and altered inflammatory signaling [4, 5]. This process weakens barrier integrity, inhibits basal cell proliferation, postpones tissue repair, and maintains inflammatory reactions that worsen tissue damage over time [6].

The benefits of current clinical interventions are only partially realized. Hydrocolloid dressings, moisturizers, topical corticosteroids, and other supportive treatments alleviate symptoms but do not successfully address the intracellular processes causing tissue deterioration [7, 8]. The only FDA-approved radioprotector, Amifostine, is restricted due to systemic toxicity and limited suitability for topical use. As a result, there is a clear gap in therapeutic development [9, 10]. Despite growing global interest in nanoparticle-based therapies and an increasing understanding of the molecular pathways underlying radiation-induced injury, relatively limited research has focused on developing clinically translatable and biocompatible topical nano-systems capable of delivering antioxidant activity directly to the intracellular environment of damaged skin [11].

Hesperidin (HSP) is a citrus flavonoid that possesses strong antioxidant, anti-inflammatory, and wound healing activity. It has been shown to attenuate ROS generation, restore barrier function, and modulate NF kappa B, iNOS and related molecular pathways involved in oxidative and inflammatory injury [12, 13]. These properties make HSP a promising therapeutic candidate for managing radiation-induced skin damage. However, HSP is a Biopharmaceutics Classification System class IV compound with low aqueous solubility, limited permeability, and poor dermal penetration [14]. Its access to the viable epidermis/dermis, where radiation-induced oxidative stress takes place, is limited by these physicochemical constraints. HSP is commercially available primarily as oral dietary supplements and in combination products (e.g., with diosmin), typically in doses ranging from 250 to 500 mg. However, no clinically established topical formulations are available, limiting its direct application for dermal conditions. While a number of lipid and polymer-based formulations have made an effort to enhance topical HSP delivery, none have been able to effectively address the combined difficulties of intracellular delivery, permeability, and solubility [15–17]. Because there isn’t a suitable carrier that can effectively deliver the compound to radiation-affected skin cells, HSP’s therapeutic potential is still unrealized.

Solid lipid nanoparticles (SLNs) offer a practical approach for improving the topical delivery of compounds with limited aqueous solubility. These nanoscale carriers can incorporate lipophilic drugs into a stable lipid matrix and are made of physiologically compatible lipids that stay solid at body temperature [18]. SLNs can enhance drug solubilization, promote penetration into the stratum corneum, and increase residence time within the skin through occlusive effects that improve hydration and reduce transepidermal water loss [19]. Their nanoscale size allows for controlled and prolonged release of the encapsulated medication as well as close contact with skin lipids. When taken as a whole, these characteristics make SLNs appealing carriers for topical antioxidant delivery that tends to act within radiation-damaged skin [20].

Despite these benefits, conventional SLNs mainly improve drug penetration and retention within the skin and do not specifically address limitations related to intracellular drug availability. In radiation-induced skin injury, the key pathological processes, such as excessive ROS production, inflammatory responses, and cellular damage, take place within viable skin cells. For this reason, delivery systems that primarily enhance superficial penetration may not be sufficient to fully utilize the therapeutic potential of antioxidant compounds like HSP. This limitation points to a clear gap in current topical nanocarrier approaches for the treatment of radiation-induced skin injury [21].

Hyaluronic acid (HA) offers a biologically relevant means of overcoming this shortcoming. HA is a polysaccharide that is produced naturally in the extracellular matrix and constitutes the major part of it, with which it contributes significantly to barrier repair and wound healing [22, 23]. HA, when utilized as a surface modifier of nanocarrier, has been reported to enhance contact with the damaged epidermal tissue, increase retention in the non-hydrated skin, and residence in the hydrated and inflamed environment [24, 25]. HA is found in topical systems to enhance skin hydration, bioadhesion, and extended localization of formulations in the epidermis and dermis, and these are all desirable in the management of radiation-induced skin injury [26, 27]. HA functionalization of lipid nanoparticles has been reported to improve topical performance by promoting closer association with the skin surface, facilitating diffusion into hydrated skin layers, and supporting sustained drug availability at the site of injury [28, 29]. Importantly, these effects can be achieved without altering the internal structure of the lipid matrix or compromising drug encapsulation efficiency. While HA-modified nanocarriers have been explored in other dermatological and inflammatory contexts, there are currently no reports describing HA-functionalized SLNs for the topical delivery of HSP in radiation-induced skin injury.

Building on these considerations, the present study proposes the development of HA-functionalized HSP-loaded SLNs as an advanced topical delivery system for the management of radiation-induced skin injury. The central hypothesis of this study is that HA-coated SLNs can overcome the solubility and permeability limitations of HSP, improve its penetration and retention within the skin, and support sustained intracellular antioxidant activity through increased local availability in skin tissue. To evaluate this hypothesis, a Central Composite Design (CCD)-based optimization approach was used to develop a formulation with high entrapment efficiency and a particle size below 300 nm, a range considered suitable for skin delivery while limiting systemic translocation. Differential scanning calorimetry, X-ray diffraction, and transmission electron microscopy were used to confirm drug encapsulation and solid-state transformation within the lipid matrix. Ex vivo skin permeation studies were performed to quantify drug deposition and transport across full-thickness skin, and a human PBMC model was used to assess the formulation’s ability to attenuate intracellular ROS under PMA-induced oxidative stress. The work presented here proposes a HA-functionalized SLN platform as a promising approach to improve delivery in the setting of radiation-induced skin injury. The combined features of HA–HSP-SLN including enhanced skin interaction, nanoscale penetration, and sustained drug release may help overcome several biological and pharmaceutical challenges that limit the topical utility of free hesperidin. While further studies will be required to establish clinical relevance, the findings provide a rational foundation for advancing antioxidant nanocarriers as potential candidates for future development in the management of radiation-related skin toxicity.

## Materials and Methods

### Materials

HSP was obtained from Yarrowchem Products (India). Compritol^®^ 888 ATO was received as a gift sample from Gattefosse India. Transcutol^®^ HP was procured from Tokyo Chemical Industry (India). Poloxamer^®^ 407 was purchased from Sigma–Aldrich (USA). HA and soya lecithin were sourced from SRL Pvt. Ltd. (India). All other chemicals and reagents were of analytical grade and supplied by SD Fine-Chem (India).

### Analytical Method

HSP was quantified using a previously validated HPLC method [30]. Analyses were performed on a Shimadzu i-Series LC-2050 C 3D system (Shimadzu, Japan) equipped with a photodiode array (PDA) detector and autosampler. Chromatographic separation was achieved on an HSS C18 column (250 × 4.0 mm, 5 μm). The mobile phase consisted of methanol and water (50:50 v/v) under isocratic conditions at 40 °C, with a flow rate of 0.9 mL/min and an injection volume of 10 µL. Detection was conducted at 280 nm using LabSolutions software (version 5.106) for data acquisition and processing, as reported previously.

### SLN Preparation

SLNs were prepared using the solvent injection – hot homogenization method. The aqueous phase was prepared by dispersing Polysorbate 80 and Poloxamer 407 in distilled water. The lipid phase, comprising Compritol^®^ 888 ATO, Transcutol^®^ HP and soya lecithin, was dissolved in an ethanol–chloroform mixture (1:1 v/v). HSP was dissolved in the lipid phase at a concentration of 0.2% w/v of dispersion. Both phases were heated to 80 °C, and the organic phase was introduced dropwise into the aqueous phase under continuous stirring. The resulting coarse dispersion was subjected to high-speed homogenization (Silent Crusher M, Heldolph Instruments, USA) at 15,000 rpm for 10 min, followed by probe sonication (VC-750, Sonics and Materials Inc., USA) for an additional 10 min to obtain uniform nanoemulsion. The hot nanoemulsion was then allowed to cool to room temperature to form SLNs [31, 32]. HA was added to the SLN dispersion at an optimized concentration of 0.2% w/w and stirred magnetically for 2 h. This step enabled non-covalent adsorption of HA onto the nanoparticle surface, likely driven by hydrogen bonding and interfacial interactions with surfactant-rich domains [33]. This post-coating step yielded HA-coated HSP-loaded SLNs (HSP-HA-SLN). Fourier-transform infrared (FTIR) spectroscopy was performed to assess chemical compatibility and confirm HA coating of the SLN formulations (Supplementary Information).

### SLNs Optimization Using Design of Experiment (DoE)

Formulation optimization was performed using a Central Composite Design (CCD) to examine the effects of key formulation variables on SLN performance. The CCD approach enabled systematic variation of factors using factorial, axial, and center points, allowing efficient exploration of the formulation design space [34]. Lipid concentration (X_1_) and surfactant concentration (X_2_) were selected as independent variables, while particle size and entrapment efficiency were defined as the response variables. Table 1 details the design matrix for the optimization of SLNs. Response surface modelling was used to establish quantitative relationships between formulation components and measured outputs, and to identify optimal conditions for achieving the desired SLN characteristics [35, 36]. Statistical evaluation of model parameters was performed to determine the best-fitting polynomial equation and to ensure adequate predictive capability. Numerical and graphical optimization were employed for identifying the optimized composition. The formulation was optimized on the basis of the desirability function from the identified design space in the overlay plot and within the set constraints for the response variables.

**Table 1:** Design matrix for optimization of SLN

Independent variables/levels of Independent variables	Lipid Concentration (%, X_1_)	Surfactant Concentration (%, X_2_)	Dependent Variables	Constraints for dependent variables
High	2	1	Particle Size (nm)	Minimize (200–400 nm)
Centre	3	2	EE (%)	Maximize (more than 80%)
Low	4	3		
Batches	**Lipid Concentration (%**,** X**_**1**_**)**	**Surfactant Concentration (%**,** X**_**2**_**)**	**Particle Size (nm)**	**EE (%)**
1	3	2	282 ± 3.4	85.3 ± 2.4
2	3	2	282.1 ± 2.5	83.1 ± 3.1
3	2	3	251.6 ± 4.5	77.45 ± 4.3
4	4	1	986.6 ± 7.8	71.24 ± 1.9
5	3	3.41	450.3 ± 2.3	83.19 ± 2.7
6	4	3	469.4 ± 6.7	79.16 ± 3.8
7	3	0.58	1109.9 ± 9.8	77.45 ± 2.9
8	4.41	2	527 ± 6.3	74.13 ± 1.1
9	3	2	285.4 ± 3.5	85.1 ± 2.3
10	3	2	290 ± 3.4	85.5 ± 3.4
11	2	1	463.2 ± 4.5	72.8 ± 4.2
12	3	2	282.8 ± 3.5	82.2 ± 3.9
13	1.58	2	269.5 ± 3.4	68 ± 2.6

### Lyophilization

The SLN dispersion was freeze-dried using a lyophilizer (Ishin Biobase, South Korea). Samples were pre-frozen in vials and subjected to a 24-hour automated lyophilization cycle operated at a chamber pressure of 5 mBar and a condenser temperature of -80 °C.

### Characterization of Optimized HSP-HA-SLN

#### Particle Size and Zeta Potential

Particle size and zeta potential of the Blank SLN, HSP-SLN and HSP-HA-SLN were measured using a Zetasizer Nano ZS-90 (Malvern Instruments, UK). Measurements were performed after diluting the sample 10 folds with HPLC grade water at 25 ± 1 °C with a scattering angle of 130°. For each formulation, the mean value and standard deviation were calculated from three independent determinations [37, 38].

#### Entrapment Efficiency and Drug Loading

Entrapment efficiency (EE) and Drug Loading (DL) was determined by ultracentrifugation at 15,000 rpm for 30 min at 4 °C using a refrigerated centrifuge (Remi, India). The supernatant was collected and appropriately diluted with methanol. The amount of unentrapped (free) drug in the supernatant was quantified by HPLC, and EE was calculated using the following equation:

$$\:\mathrm{E}\mathrm{E}\:\left({\%}\right)=\:\frac{(\mathrm{T}\mathrm{o}\mathrm{t}\mathrm{a}\mathrm{l}\:\mathrm{d}\mathrm{r}\mathrm{u}\mathrm{g}-\mathrm{F}\mathrm{r}\mathrm{e}\mathrm{e}\:\mathrm{d}\mathrm{r}\mathrm{u}\mathrm{g})\text{}}{\mathrm{T}\mathrm{o}\mathrm{t}\mathrm{a}\mathrm{l}\:\mathrm{d}\mathrm{r}\mathrm{u}\mathrm{g}}\:\times\:100$$

$$\:\mathrm{D}\mathrm{L}\:\left({\%}\right)=\:\frac{\mathrm{E}\mathrm{n}\mathrm{t}\mathrm{r}\mathrm{a}\mathrm{p}\mathrm{p}\mathrm{e}\mathrm{d}\:\mathrm{d}\mathrm{r}\mathrm{u}\mathrm{g}\text{}}{\mathrm{S}\mathrm{o}\mathrm{l}\mathrm{i}\mathrm{d}\:\mathrm{L}\mathrm{i}\mathrm{p}\mathrm{i}\mathrm{d}}\:\times\:100$$

#### High-resolution Transmission Electron Microscopy (TEM)

The SLN dispersion was diluted 1:100 and stained with 1% phosphotungstic acid. A small volume of the sample was placed onto a copper grid, and excess fluid was blotted off before air drying in a dust-free environment. Imaging was performed using a Technai 20 TEM (Philips) operated at an accelerating voltage of 100 kV in bright-field mode, with a nominal magnification of 28,500× [39].

#### Differential Scanning Calorimetry

Differential scanning calorimetry (DSC) was performed using a DSC Q600 instrument (TA Instruments, USA). Approximately 10 mg of each sample (HSP and HSP-HA-SLN) was sealed in aluminum pans and heated from 25 °C to 300 °C at a rate of 10 °C/min under a nitrogen purge of 20 mL/min. An empty aluminum pan served as the reference [40].

#### X-ray Diffraction (XRD) Analysis

The crystallinity of HSP and HSP-HA-SLN was evaluated using powder XRD. Measurements were performed on a D6 PHASER diffractometer (Bruker India Scientific Pvt. Ltd., USA) equipped with nickel-filtered Cu Kα radiation (40 kV, 30 mA). Samples were scanned over a 2θ range of 2° to 70° with a step size of 0.026°, at a controlled temperature of 25 °C.

### Functional Assessment

#### In Vitro Drug Release Study

The release profile of HSP-HA-SLN was evaluated using a modified Franz diffusion cell with an effective diffusion area of 2.54 cm². A pre-activated dialysis membrane (MWCO 12–14 kDa, Himedia, India) was mounted between the donor and receptor compartments. An SLN dispersion equivalent to 10 mg of HSP was placed in the donor chamber. The receptor compartment contained phosphate-buffered saline (pH 7.4) with 10% methanol, maintained at 32 °C and agitated continuously with slow-speed magnetic stirring. At predetermined intervals, aliquots were withdrawn and replaced with fresh medium to maintain sink conditions. HSP content in the samples was quantified by HPLC, and cumulative release was expressed as a percentage of the total drug loaded [41].

#### Cytotoxicity Assay

The cytotoxicity of HSP, blank SLN and HSP-HA-SLN was assessed in human epidermoid carcinoma A431 cells using the MTT assay. Cells were cultured in Dulbecco’s Modified Eagle Medium supplemented with 10% fetal bovine serum, 100 U/mL penicillin and 100 µg/mL streptomycin and maintained at 37 °C in a humidified atmosphere containing 5% CO₂. Cells were seeded at a density of 1 × 10⁴ cells per well in a 96-well plate and allowed to adhere overnight. Formulations were then added at concentrations of 100, 50, 25, 12.5, 6.25 and 3.125 µg/mL, followed by incubation for 48 h. After washing twice with PBS, 20 µL of MTT solution was added to each well and incubated for 4 h at 37 °C. Formazan crystals were dissolved by adding 100 µL of DMSO, and absorbance was measured at 570 nm using a microplate reader [42]. Cell viability was calculated using the formula:

$$\:\mathrm{C}\mathrm{e}\mathrm{l}\mathrm{l}\:\mathrm{v}\mathrm{i}\mathrm{a}\mathrm{b}\mathrm{i}\mathrm{l}\mathrm{i}\mathrm{t}\mathrm{y}\:\left({\%}\right)=\:\frac{\mathrm{M}\mathrm{e}\mathrm{a}\mathrm{n}\:\mathrm{O}\mathrm{D}\:\mathrm{o}\mathrm{f}\:\mathrm{t}\mathrm{e}\mathrm{s}\mathrm{t}\:\mathrm{s}\mathrm{a}\mathrm{m}\mathrm{p}\mathrm{l}\mathrm{e}\text{}}{\mathrm{M}\mathrm{e}\mathrm{a}\mathrm{n}\:\mathrm{O}\mathrm{D}\:\mathrm{o}\mathrm{f}\:\mathrm{n}\mathrm{e}\mathrm{g}\mathrm{a}\mathrm{t}\mathrm{i}\mathrm{v}\mathrm{e}\:\mathrm{c}\mathrm{o}\mathrm{n}\mathrm{t}\mathrm{r}\mathrm{o}\mathrm{l}}\:\times\:100$$

#### Antioxidant Efficacy

Peripheral blood mononuclear cells (PBMCs) were isolated from 10 mL of peripheral blood obtained from healthy volunteers after ethical approval and written informed consent (PUIECHR/PIMSR/00/081734/7212). Isolation was performed using a density gradient centrifugation protocol. Whole blood was centrifuged at 2000 rpm for 10 min to separate plasma, after which the buffy coat was subjected to dextran sedimentation in HBSS for 30 min. The supernatant was centrifuged at 2000 rpm for 5 min, and the resulting pellet was resuspended in HBSS and layered over Histopaque (1119/1077). Following centrifugation at 700 g for 15 min with minimal acceleration and deceleration, PBMCs were collected from the interface, washed with HBSS, and resuspended in RPMI medium [43]. To evaluate intracellular ROS generation, PBMCs were seeded in RPMI and stimulated with phorbol-12-myristate-13-acetate (PMA, 50 nM) [44, 45]. Cells were then treated with blank SLN, free HSP or HSP-HA-SLN formulations to a final reaction volume of 300 µL. After 2 h of incubation at 37 °C, intracellular ROS were quantified using 2′,7′-dichlorofluorescein diacetate (DCF-DA), and fluorescence was measured using a multimode fluorescence reader [46]. The stimulation index was calculated relative to unstimulated control cells, and results were expressed as mean ± SD.

#### Ex Vivo Drug Permeation and Retention Study

Animal experiments were conducted in accordance with CPCSEA guidelines and approved by the Institutional Animal Ethics Committee (Protocol No. PIPR 984/2024/02/19). Full-thickness abdominal skin was excised from healthy Albino Wistar rats (290–300 g). Abdominal hair was carefully trimmed to preserve skin integrity, after which animals were humanely euthanized, and the excised skin was cleaned by removal of subcutaneous fat. The prepared skin was mounted on Franz diffusion cells with an effective diffusion area of 2.54 cm², positioning the stratum corneum facing the donor compartment. HSP-HA-SLN and uncoated HSP-SLN dispersions, each equivalent to 10 mg of HSP, were separately applied using a micropipette to the donor compartment to enable comparative evaluation of permeation and skin retention. The receptor compartment was filled with 25 mL of phosphate buffer (pH 7.4) containing 10% methanol, maintained at 32 ± 0.5 °C and stirred continuously at 50 rpm. Samples were collected at predetermined time points and analyzed to determine cumulative drug permeation [47, 48]. After 24 h, the skin was removed from the diffusion cells, cut into small pieces, and immersed in methanol. The tissue was then homogenized and subjected to bath sonication for 30 min to extract the drug retained within the skin layers. The extracts were filtered through a 0.45 μm membrane filter, and the HSP content was quantified by HPLC [49]. This comparison was carried out to clearly assess the contribution of HA surface functionalization to skin permeation and retention relative to the uncoated SLN system.

### Statistical Analysis

All experiments were performed in triplicate unless stated otherwise. Data analysis was carried out using GraphPad Prism (GraphPad Software, USA). Results are reported as mean ± standard deviation (SD). Statistical significance was evaluated using one-way ANOVA followed by appropriate post hoc tests, with *p* < 0.05 taken as statistically significant.

## Results

### Formulation Development and Analytical Method

HSP quantification in all formulations was carried out using a previously developed and validated HPLC method reported by our group. This method was used throughout the study to measure drug content, entrapment efficiency, release behavior, and permeation profiles. HSP-loaded SLNs were prepared using a solvent injection- hot homogenization method, in which a nanoemulsion was formed at elevated temperature and then cooled to allow solidification of lipid droplets into nanoscale particles. Addition of HA to the SLN dispersion resulted in the formation of HSP-HA-SLN.

### SLNs Optimization Using DoE

SLN optimization was carried out using a Central Composite Design (CCD) within a Quality-by-Design framework to examine the effects of lipid concentration (X₁) and surfactant concentration (X₂) on particle size (Y₁) and entrapment efficiency (Y₂). Thirteen experimental runs were generated, including four factorial points, four axial points (±α = 1.414), and five center-point replicates. This design allowed systematic exploration of the formulation space while accounting for potential curvature effects. Table 1 outlines the independent variables, measured responses, applied constraints, and experimental runs. A wide variation was observed in particle size across the design space, ranging from 251.6 ± 4.5 nm to 1109.9 ± 9.8 nm, emphasizing the strong influence of formulation variables. Entrapment efficiency ranged from 68.0 ± 2.6% to 85.5 ± 3.4%. Notably, center-point formulations consistently produced particle sizes in the range of approximately 280–290 nm and entrapment efficiencies above 82%, demonstrating good experimental reproducibility and formulation robustness.

Quadratic polynomial models were identified as the most suitable for describing both responses, with minimal lack of fit. The regression equations derived for particle size and entrapment efficiency were as follows:

- Y₁ (Particle size, nm) = 282.00 + 138.26X₁ − 207.60X₂ − 76.10X₁X₂ + 237.16X₂²
- Y₂ (Entrapment efficiency, %) = 85.30 + 1.11X₁ + 2.59X₂ − 7.25X₁² − 2.62X₂²

Statistical validation of the models is presented in Table 2. The adjusted R² values were 0.9505 for particle size and 0.9554 for entrapment efficiency, indicating excellent model fit. Predicted R² values of 0.7948 and 0.8149, respectively, were in reasonable agreement with adjusted R² values, confirming good predictive performance. Adequate precision values exceeded 18 for both responses, demonstrating an acceptable signal-to-noise ratio. Low coefficients of variation further indicated consistency across experimental runs.

**Table 2:** Statistical validation parameters for CCD-derived quadratic models describing the effect of formulation variables on SLN characteristics

Response (Dependent Variable)	Adjusted *R*²	Predicted *R*²	Adequate Precision	Coefficient of Variation (%)
Particle size (nm)	0.9505	0.7948	20.5735	3.64
Entrapment efficiency (%)	0.9554	0.8149	18.0065	1.66

**Fig. 1:**
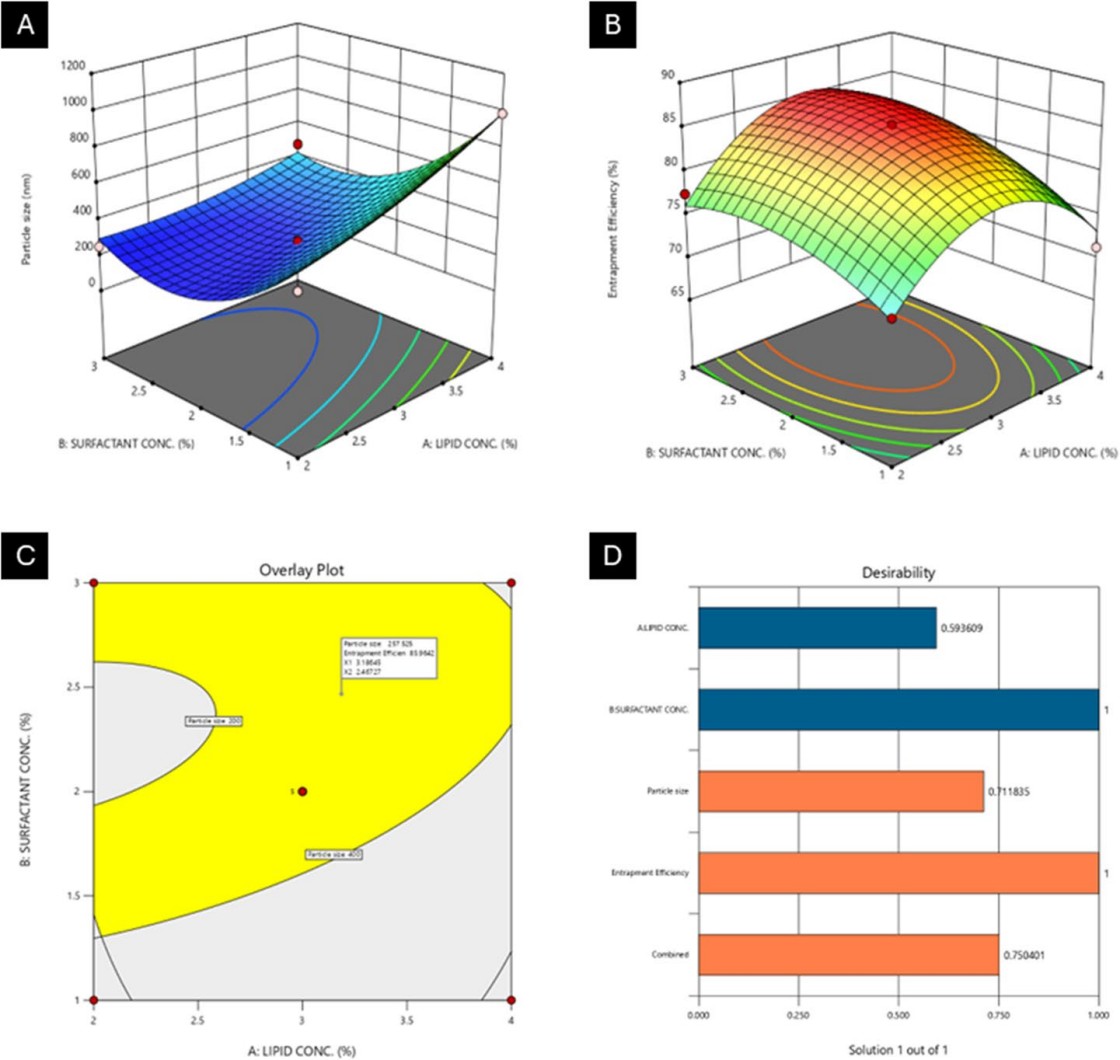
Response surface and optimization plots for DoE-based preparation of SLNs. (**A**) Three-dimensional response surface plot illustrating the combined effect of lipid concentration (X₁) and surfactant concentration (X₂) on particle size. (**B**) Three-dimensional response surface plot showing the influence of lipid and surfactant concentrations on entrapment efficiency. (**C**) Overlay plot defining the design space that simultaneously satisfies the predefined constraints for particle size (200–400 nm) and entrapment efficiency (> 80%), with the optimal formulation region highlighted. (**D**) Desirability plot depicting individual and overall desirability values for formulation variables and responses, indicating the optimized SLN composition selected for further studies

Three-dimensional response surface plots illustrating the effects of lipid and surfactant concentrations on particle size and entrapment efficiency are shown in Fig. 1a and b, respectively. The overlay plot identifying the optimal design space that met both response constraints is shown in Fig. 1c, while the overall desirability profile is presented in Fig. 1d. Numerical optimization based on desirability criteria predicted an optimal formulation containing 3.18% lipid and 2.46% surfactant, with a predicted particle size of 257.6 nm and an entrapment efficiency of 85.9%. The overall desirability value for this formulation was 0.75. Its position near the center of the design space indicates good robustness to small formulation changes and supports its selection for further physicochemical characterization and biological evaluation [50–52].

### Characterization of Optimized HSP-HA-SLN

**Fig. 2:**
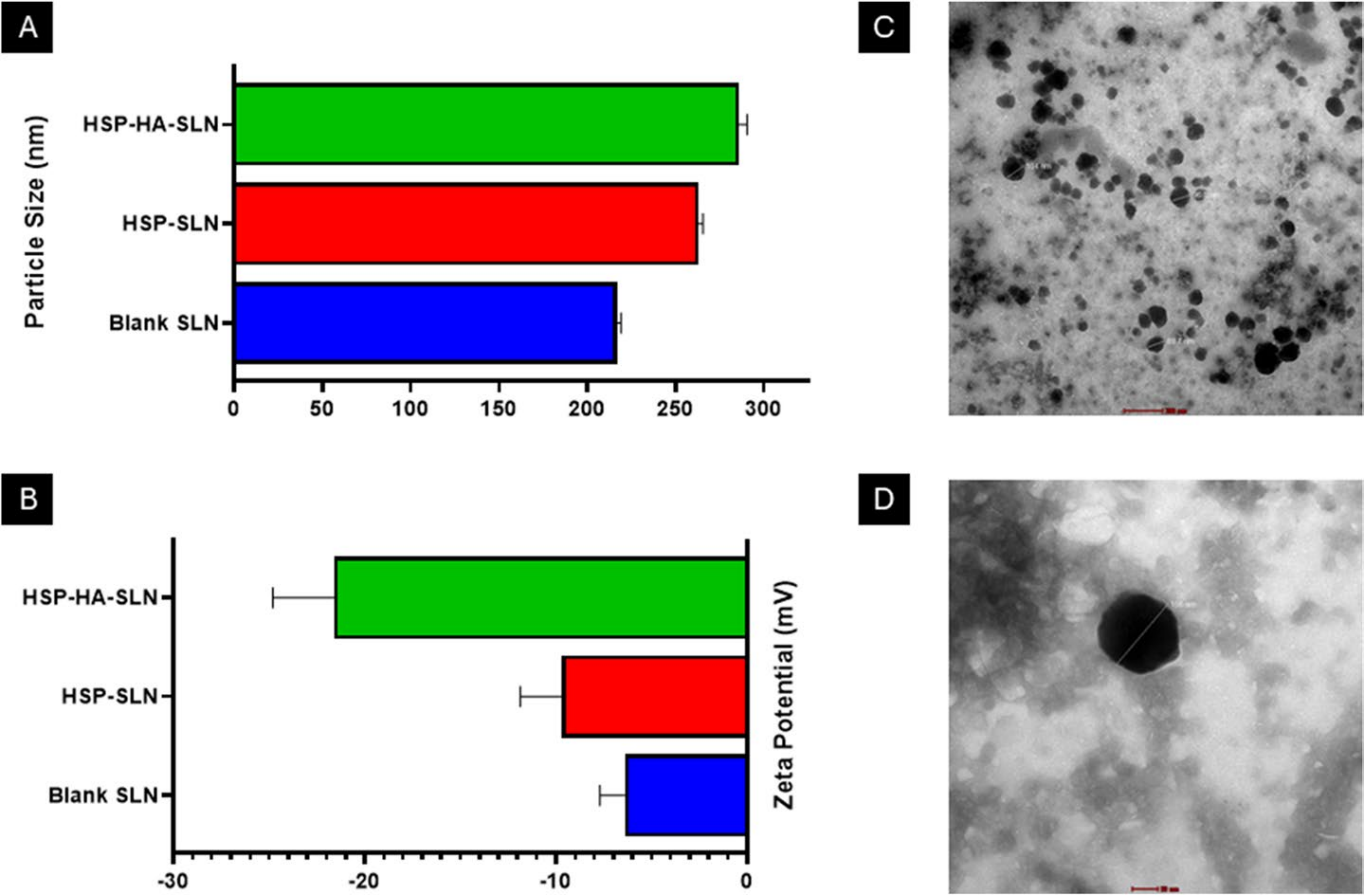
Particle size, zeta potential, and morphological characterization of SLNs. (**A**) Mean particle size of blank SLN, HSP-SLN, and HSP-HA-SLN, showing a progressive increase in size following drug loading and HA functionalization. (**B**) Zeta potential of blank SLN, HSP-SLN, and HSP-HA-SLN, demonstrating a marked increase in negative surface charge after HA coating, indicative of successful surface functionalization. (**C**) HR-TEM micrograph of optimized HSP-HA-SLN at lower magnification, revealing spherical, well-dispersed nanoparticles with narrow size distribution (Scale bar: 200 nm). (**D**) Higher-magnification HR-TEM image of an individual HSP-HA-SLN particle, showing a dense lipid core with a faint peripheral region corresponding to the HA coating layer (Scale bar: 50 nm)

Particle size and zeta potential of blank SLN, HSP-SLN, and the optimized HSP-HA-SLN were measured, and the results are shown in Fig. 2A-B. Blank SLNs had a mean particle size of 217 ± 2.11 nm. Loading HSP into the lipid matrix increased the particle size, with HSP-SLN showing a mean diameter of 263 ± 2.55 nm. Further surface modification with HA led to a moderate additional increase in size, giving a final mean diameter of 286 ± 4.5 nm for HSP-HA-SLN. Zeta potential measurements showed a gradual increase in negative surface charge with each formulation step. Blank SLNs exhibited a zeta potential of − 6.4 ± 1.3 mV, which became slightly more negative after drug loading (HSP-SLN: −9.7 ± 2.15 mV). Functionalization of HA caused a significant change of surface charge where HSP-HA-SLN exhibited a zeta potential of -21.6 ± 3.2 mV. The entrapment efficiency of the optimized HSP-HA-SLN formulation was found to be 83.2 ± 3.4%. The drug loading was found to be 5.23% w/w with respect to solid lipid and considering the entrapment efficiency. No statistically significant difference in entrapment efficiency was observed between HA-coated and uncoated HSP-SLNs, indicating that surface functionalization did not adversely affect drug loading within the lipid matrix. HR-TEM images of the optimized HSP-HA-SLN formulation are shown in Fig. 2C–D. The nanoparticles appeared predominantly spherical, well dispersed, and uniform in morphology, with no evidence of aggregation. The particle sizes observed by TEM were smaller than those measured by dynamic light scattering. At higher magnification, individual particles displayed a dense core surrounded by a faint peripheral region [53]. Comparative FTIR analysis (Figure S1) supported component compatibility and HA-coated SLN formation.

**Fig. 3:**
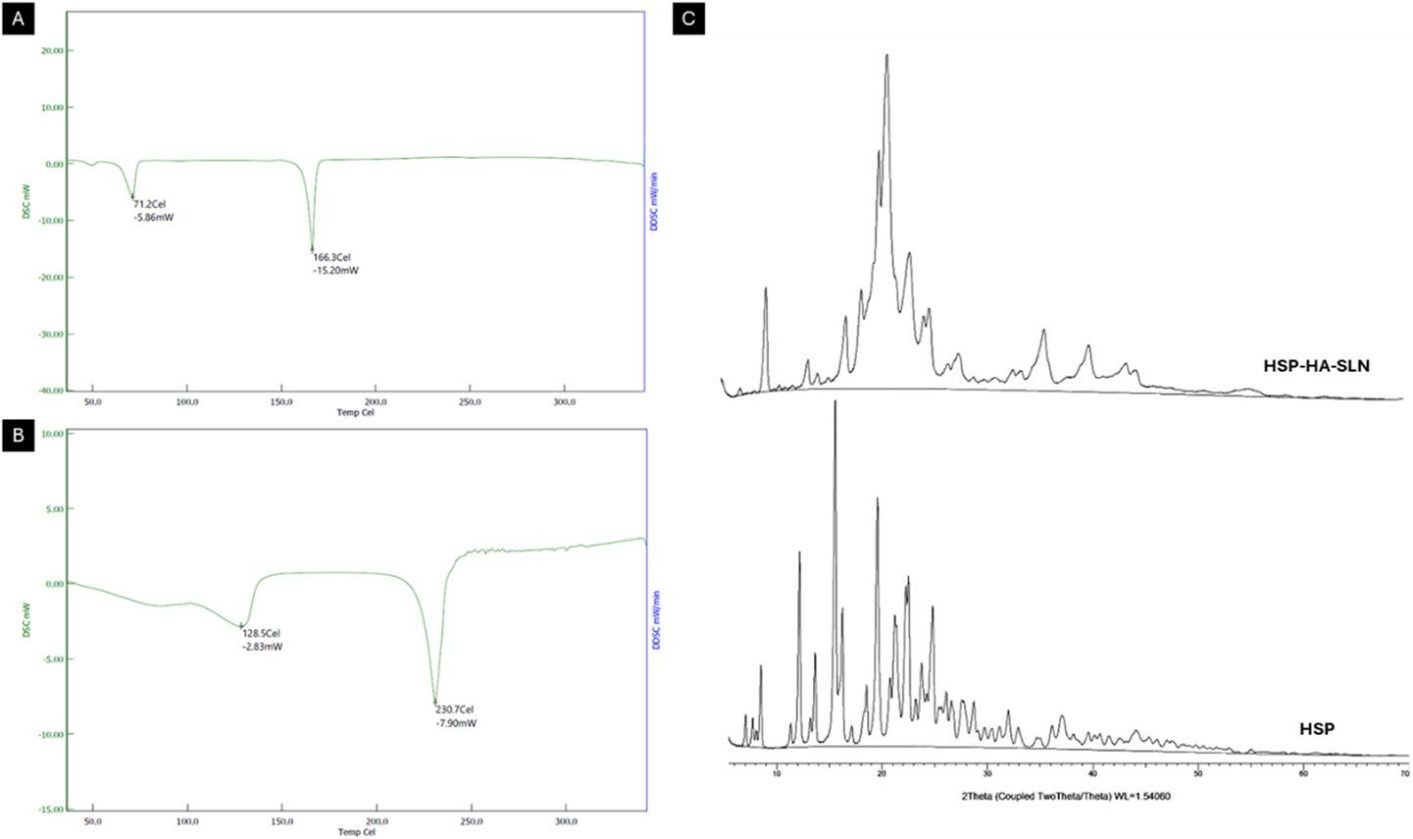
DSC thermograms of (**A**) pure HSP and (**B**) optimised HSP-HA-SLN, showing disappearance of the crystalline HSP melting endotherm and the characteristic melting peak of glyceryl behenate. (**C**) X-ray diffraction patterns of pure HSP and optimised HSP-HA-SLN, demonstrating loss of drug crystallinity and formation of an amorphous or molecularly dispersed state within the lipid matrix

DSC was employed to evaluate the thermal behavior of pure HSP and the optimized HSP-HA-SLN formulation. The DSC thermograms are presented in Fig. 3A-B. Pure HSP exhibited a sharp endothermic peak in the temperature range of 252–261 °C, consistent with its reported melting point and confirming its highly crystalline nature. In addition, a minor endothermic event was observed at approximately 128 °C, which may be attributed to the loss of loosely bound moisture or residual solvent. These features collectively indicate a chemically pure and crystalline drug substance. In contrast, the DSC thermogram of the optimized HSP-HA-SLN formulation showed a distinct endothermic peak at approximately 71 °C, corresponding to the melting of the lipid matrix, glyceryl behenate (Compritol^®^ 888 ATO). The characteristic melting endotherm of crystalline HSP was absent in the SLN thermogram [54, 55]. A new endothermic transition was observed around 166–170 °C. No exothermic peaks associated with oxidation or thermal degradation were detected below 300 °C, indicating thermal stability of the formulation under the applied conditions. Powder X-ray diffraction analysis was performed to further investigate the solid-state characteristics of HSP and the optimized HSP-HA-SLN. The diffractograms are shown in Fig. 3C. Pure HSP displayed numerous sharp and intense diffraction peaks across the 2θ range of approximately 8–30°, confirming its highly crystalline structure. In contrast, the XRD pattern of the optimized HSP-HA-SLN exhibited a marked reduction in peak intensity, with the absence of the characteristic crystalline peaks of HSP. The diffractogram was dominated by broadened, low-intensity reflections and a diffuse halo pattern typical of lipid-based matrices [17].

### Functional Assessment

#### In Vitro Drug Release Behavior

**Fig. 4:**
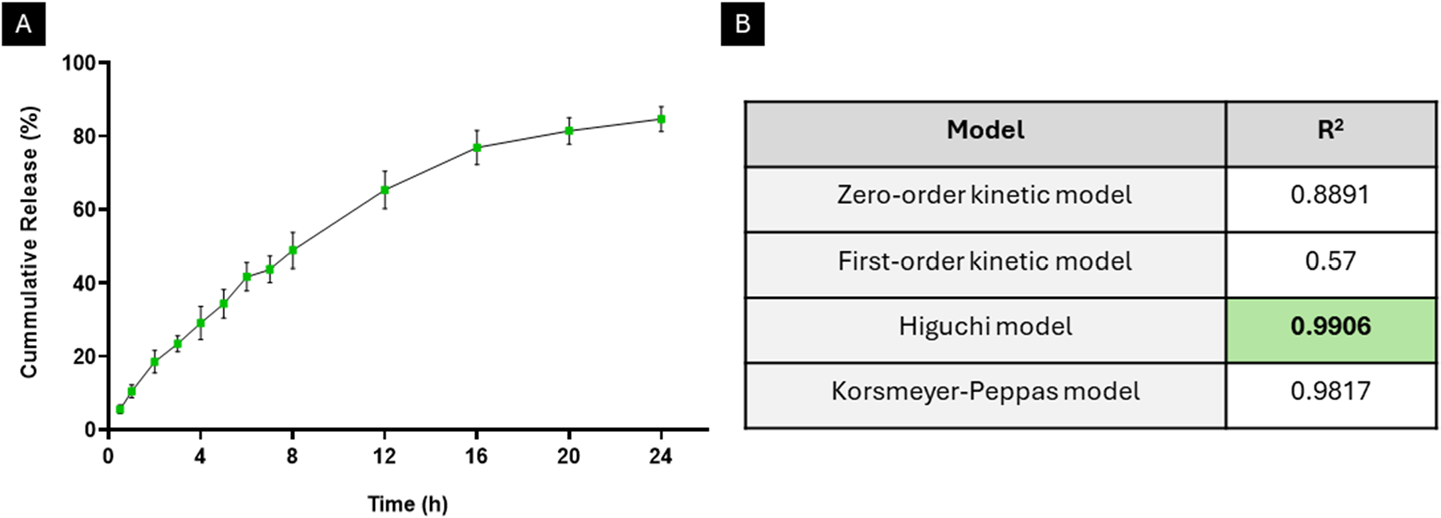
In vitro drug release behaviour and kinetic modelling of optimised HSP-HA-SLN. (**A**) Cumulative release profile of HSP from HSP-HA-SLN, demonstrating a sustained and controlled release pattern over time. (**B**) Summary of kinetic modelling applied to the release data, showing the highest correlation with the Higuchi model (R² = 0.9906), indicating diffusion-controlled drug release from the solid lipid matrix

The in vitro release profile of HSP from the optimized HSP-HA-SLN formulation was evaluated over 24 h using a modified Franz diffusion system (Fig. 4A). The formulation exhibited a gradual, sustained release pattern with no evidence of an initial burst, indicating efficient drug encapsulation within the solid lipid matrix. The formulation exhibited a gradual and sustained release pattern over 24 h, with no pronounced initial burst, indicating efficient encapsulation of HSP within the solid lipid matrix. Only 10.44 ± 1.80% of the drug was released till 1 h, implying minimal surface-associated drug and absence of burst release. Cumulative drug release increased with time, reaching 23.46 ± 2.20% at 3 h and 41.68 ± 3.80% at 6 h. This indicated a sustained diffusion phase, with drug release of 48.81 ± 4.90% at 8 h and 65.29 ± 5.10% at the end of 12 h. By 16 h, 76.85 ± 4.70% of HSP had been released, and a maximum cumulative release of 84.67 ± 3.40% was observed at 24 h, indicating controlled and reproducible release pattern (*n* = 6) [56]. To assess the release mechanism, the data were fitted to different kinetic models (Fig. 4B). The Higuchi model showed the highest correlation coefficient (R² = 0.9906), suggesting that HSP release was mainly governed by Fickian diffusion through the solid lipid matrix. The Korsmeyer–Peppas model also demonstrated a strong fit (R² = 0.9817), supporting diffusion-dominated transport with minimal contribution from matrix erosion or swelling. The Higuchi model suggested the probable release kinetics while Korsmeyer–Peppas model gave mechanistic insights into the release mechanism. In contrast, the zero-order model showed moderate correlation (R² = 0.8891), while the first-order model exhibited poor fit (R² = 0.57), ruling out concentration-dependent release kinetics [57, 58]. Overall, the numerical release profile and kinetic modeling collectively confirm that the HSP-HA-SLN formulation provides controlled, diffusion-driven, and sustained drug release over 24 h. The in-vitro release of HSP or uncoated HSP-SLN was not performed for comparison and represents the current study limitation.

#### In Vitro Cytotoxicity Evaluation

**Fig. 5:**
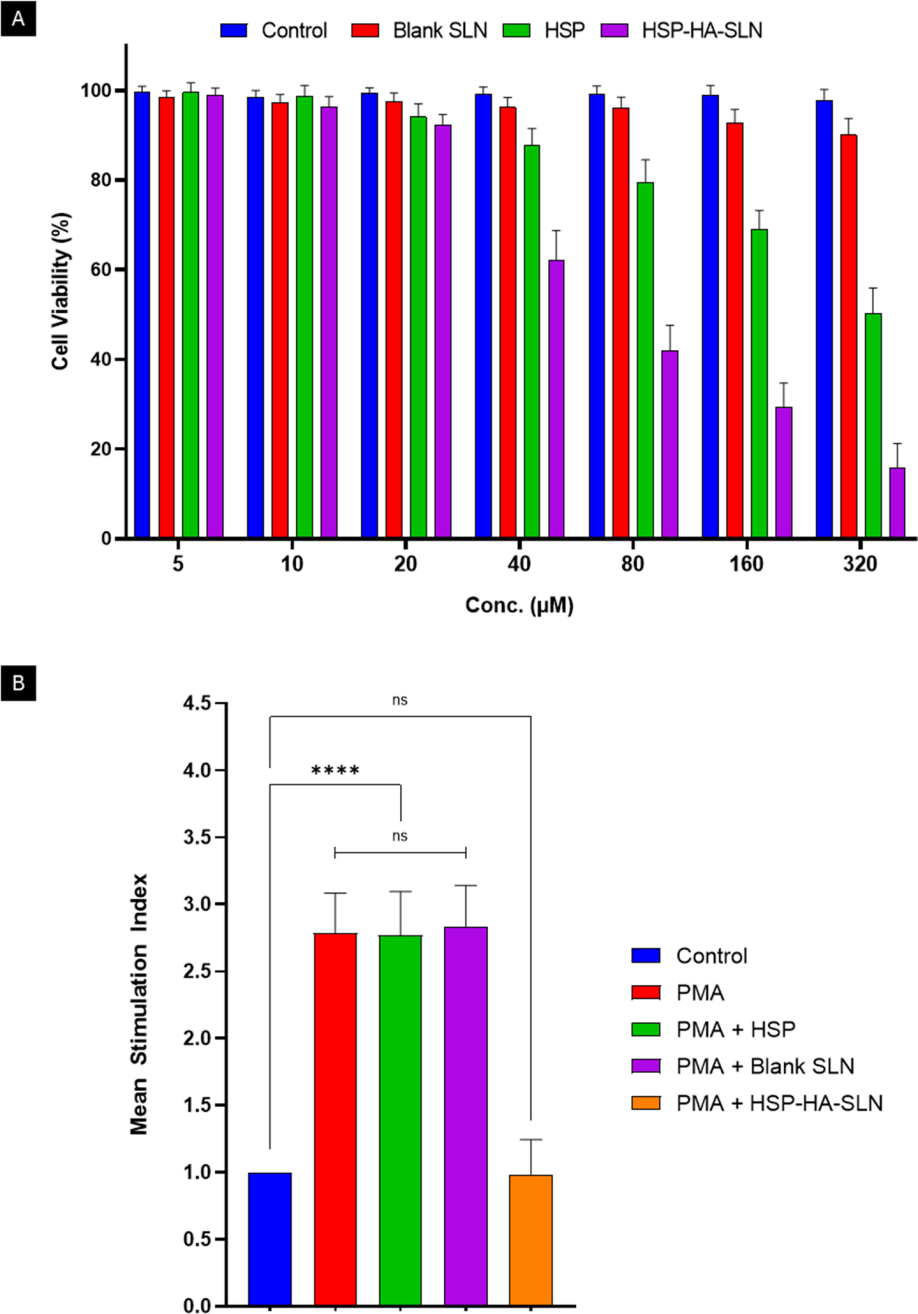
(**A**) In vitro cytotoxicity assessment of HSP-HA-SLN in A431 cells using the MTT assay after 48 h exposure. Cell viability remained high across all tested concentrations (3.125–100 µg/mL), indicating good cytocompatibility of both the carrier system and the HA-functionalized HSP-loaded formulation. (**B**) Effect of free HSP, blank SLN, and HSP-HA-SLN on PMA-induced intracellular ROS generation in human PBMCs. Treatment with HSP-HA-SLN significantly attenuated PMA-induced ROS compared with PMA alone (***p < 0.001), whereas free HSP and blank SLNs showed no significant effect (ns). Data are expressed as mean ± SD (*n* = 3)

The cytotoxicity of HSP-HA-SLN was evaluated in human epidermoid carcinoma A431 cells using the MTT assay following 48 h exposure (Fig. 5A). Untreated control cells maintained consistently high viability across the tested concentration range (97.8–99.7%), confirming assay reliability. Blank SLNs demonstrated excellent cytocompatibility, with cell viability remaining above 90% even at the highest tested concentration (320 µM; 90.1 ± 3.7%), indicating negligible carrier-associated toxicity. In contrast, both free HSP and HSP-HA-SLN exhibited a clear concentration-dependent reduction in cell viability, consistent with the reported antiproliferative and anticancer activity of HSP at elevated concentrations. At lower concentrations (5–20 µM), both formulations were well tolerated, with cell viability remaining above 92%. HSP-HA-SLN showed 99.0 ± 1.6% viability at 5 µM and 92.3 ± 2.4% at 20 µM, confirming cytocompatibility within the therapeutically relevant range for topical antioxidant applications. A pronounced reduction in cell viability was observed at concentrations of 40 µM and above. At 40 µM, cell viability decreased to 87.9 ± 5.64% for free HSP and 62.2 ± 6.5% for HSP-HA-SLN. This trend intensified at higher concentrations, with viability reducing to 79.60 ± 4.95% (free HSP) and 41.9 ± 5.7% (HSP-HA-SLN) at 80 µM, and to 50.27 ± 5.66% and 15.9 ± 5.4%, respectively, at 320 µM. These findings are in agreement with previous reports showing that HSP has the potential to inhibit cell growth and induce cytotoxic effects in carcinoma cell lines at concentrations above approximately 50 µM, mediated through mechanisms that include cell-cycle arrest, mitochondrial dysfunction, and induction of apoptosis [59, 60].

HSP-HA-SLN did not show greater cytotoxicity than free HSP when compared at the same concentrations. The modestly lower cell viability observed with HSP-HA-SLN at higher doses is most likely related to improved cellular uptake resulting from HA surface functionalization, rather than any toxic effect associated with the nanoparticle carrier itself [61, 62]. This explanation is reinforced by the higher cellular compatibility of the blank SLN formulation, which showed minimal influence on cell viability across the tested concentration range. In totality, these observations indicate that the SLN nanocarrier is inherently biocompatible. The reduction in viability observed at elevated concentrations of HSP as free drug or in formulation can be primarily attributed to the known intrinsic anticancer activity of hesperidin. The concentration-dependent nature of the cellular viability findings further advocates that HSP-HA-SLN can be used safely within the concentration range relevant for topical antioxidant therapy, while also retaining potential application for anticancer activity at higher doses.

#### Effect of HSP-HA-SLN on Intracellular ROS Production

The intracellular ROS level was determined in human PBMCs by stimulating them with PMA (50 nM), and the results were plotted as the mean stimulation index values (Fig. 5B). The mean stimulation index indicative of intracellular ROS was increased after PMA stimulation compared to the control cells. The index of PMA-induced oxidative stress rose to 2.79 ± 0.30 (*p* < 0.001 vs. control). The levels of the PMA-induced ROS were not suppressed with free HSP since the indices were comparable to PMA (2.77 ± 0.33, not significant). Blank SLNs had no effect on ROS level (non-significant 2.83 ± 0.31), and this suggests that the nanocarrier did not influence intracellular oxidative stress. Nevertheless, there was a significant decrease in the intracellular level of ROS during the treatment with HSP-HA-SLN. The stimulation index of the HSP-HA-SLN group was reduced to 0.99 ± 0.26, which was significantly lower than the PMA-stimulated cells (*p* < 0.001). ROS levels in this group were almost similar to unstimulated control cells. These results strongly indicate that HA-functionalized SLNs substantially improve the intracellular antioxidant effect of HSP, while free HSP and blank nanoparticles are not effective under the same conditions. Notably, ROS levels in the HSP-HA-SLN group were restored to near-basal values and were significantly lower than those observed in both free HSP–treated and blank SLN–treated groups. These findings demonstrate that encapsulation of HSP within the HA-functionalized SLN system is critical for achieving effective intracellular antioxidant activity.

#### Ex Vivo Drug Permeation and Retention Across Full-thickness Skin

The ex vivo permeation and skin retention behaviour of uncoated HSP-SLN and HA-functionalised HSP-HA-SLN was evaluated using full-thickness rat skin mounted on Franz diffusion cells, and the quantitative drug distribution after 24 h is summarised in Table 3. Uncoated HSP-SLN showed moderate transdermal permeation, with 33.6 ± 2.4% of the applied drug detected in the receptor compartment. Drug retention within the skin layers accounted for 22.1 ± 2.9%, while a comparatively large fraction of the drug remained unabsorbed on the skin surface (35.4 ± 2.1%). Procedural drug loss was 7.8 ± 0.8%, indicating acceptable mass balance. In contrast, HA-functionalised HSP-HA-SLN demonstrated a markedly improved distribution profile. Drug permeation into the receptor compartment increased to 48.23 ± 3.2%, representing an approximate 1.4-fold enhancement compared with uncoated SLNs. The 24 h drug permeation profile across the full thickness rat skin has been detailed in the supplementary file. Simultaneously, drug retention within the skin increased to 30.12 ± 4.2%, corresponding to a ~ 36% increase in dermal deposition. Importantly, the amount of drug remaining unabsorbed on the skin surface was markedly lower, decreasing to 14.07 ± 1.4%. This reduction suggests improved interaction between the formulation and the skin, with less drug left as surface residue. Procedural drug loss remained low at 5.68 ± 0.74% and was similar to that observed for uncoated SLNs, indicating good overall mass balance. Taken together, HA functionalisation led to a clear shift in drug distribution toward increased skin uptake and retention, while maintaining controlled permeation across the skin.

**Table 3:** Ex vivo skin permeation and retention parameters of HSP formulations after 24 h (mean ± SD, *n* = 6)

Formulation	Receptor (%)	Skin Retention (%)	Total Skin Uptake† (%)	Unabsorbed (%)	Loss (%)
HSP-SLN	33.6 ± 2.4	22.1 ± 2.9	55.7	35.4 ± 2.1	7.8 ± 0.8
HSP-HA-SLN	48.23 ± 3.2	30.12 ± 4.2	78.35	14.07 ± 1.4	5.68 ± 0.74

## Discussion

### DoE–driven Optimization and its Relevance

An important aspect of the current study is the use of Design of Experiments, specifically Central Composite Design within the Quality-by-Design framework to optimize formulation variables. These are independent variables that can impact the response variables, viz., particle size and drug entrapment efficiency. Particle size is a crucial parameter for optimization of nanocarriers, as it can affect the interaction of nanoparticles with the stratum corneum, their follicular penetration, occlusivity, as well as the risk of systemic translocation. Sub-micron particles, which are in the 200–400 nm range, are generally accepted as suitable for dermal applications. The small size allows efficient and intimate contact of nanoparticles with the stratum corneum and dermal penetration, while transgression into the systemic compartment becomes limited [63]. In the current study, the particle size of the nanoparticles was affected by both lipid and surfactant concentrations, with sizes ranging from around 250 nm to more than 1000 nm across the explored experimental space. This broad variation underscores the fact that these are critical parameters for optimization, and a change in these independent variables can produce large variations in the response variable. This also highlights the need for systematic optimization rather than trial-and-error formulation approaches.

Increasing lipid concentrations brought about an expected increase in particle size, plausibly due to the effect of melt viscosity during the emulsification process. Increased viscosity reduces the breakup of lipid droplets under homogenization and sonication shear. This translates to larger particles after cooling and solidification of lipids in the emulsion [64]. On the contrary, increasing surfactant concentration led to lower particle size, consistent with its role of lowering interfacial tension and stabilizing the emulsion droplets. However, it was observed that the quadratic effect for surfactant concentration was significant. This implies that excessive surfactant does not contribute to further size reduction. Instead, micelle formation or interfacial saturation may occur, leading to particle growth and wider distribution of particle size [65, 66]. These observations are similar to the reported SLN formulation behavior and support the aptness of the CCD model for predicting formulation outcomes.

Entrapment efficiency also displayed a parallel pattern with particle size. At lower levels of independent variables, an increase in both lipid and surfactant concentrations enhanced drug loading, probably due to a larger lipid matrix availability enabling drug incorporation and solubilization of HSP in the lipid matrix. As we go beyond the optimal range, negative quadratic effects become significant and the possibility of drug expulsion during lipid crystallization or enhanced partitioning into the aqueous phase is observed. The optimized formulation is positioned near the center of the design space and attained the desirable smaller particle size range (~ 260–280 nm) and high entrapment efficiency (> 85%). This central positioning of the optimized formulation makes it robust to minor process variations and is useful for reproducibility and scale-up.

### Physicochemical Characterization Confirms Successful Drug Encapsulation

The step-by-step increase in particle size was observed after drug loading and subsequent HA functionalization affirms the successful formation of the functionalized SLN system. Blank SLNs showed particle sizes of around 220 nm, while incorporation of HSP led to an increase in the size to approximately 260 nm, indicating drug encapsulation within the lipophilic matrix. Following HA coating, particle size further increased to about 286 nm, which is consistent with the presence of a hydrated HA layer on the nanoparticle surface, and DLS measures the hydrodynamic size [67]. Although the size increased, the size of the functionalized nanoparticles was still in a range that was deemed suitable for topical application, indicating that the HA functionalization did not affect the desired delivery essentials of the nanoparticles on the skin. The successful surface modification with HA was also supported by the use of zeta potential measurements. It was found that post-drug loading and HA coating led to progressive changes in surface charge towards the negative. Negative charge increase after HA coating is significantly high, and this could be attributed to carboxylate groups present on the backbone of HA. An increase in the magnitude of surface charge can enhance colloidal stability by enhancing electrostatic repulsions between particles, which decreases the probability of aggregation in storage and use [68]. Further, an HA-rich surface is likely to favor interactions with the skin and extracellular matrix components, which will be useful in inflamed or radiation-damaged tissue environments [69].

High-resolution TEM gave further insight into the nanoparticle surface and structure. The particles exhibited a largely spherical shape with a relatively narrow size distribution, indicating efficient emulsification and solidification during SLN preparation. Particle sizes observed by TEM were comparatively smaller than those measured by DLS, which is expected because TEM helps to visualize the dry particle core, whereas DLS reflects the hydrodynamic diameter, including the hydration layer of surface functionalized HA [70]. At higher magnifications, a faint periphery surrounding the dense lipid core was visible, which probably indicates the presence of an HA layer over the surface [71].

### Solid-state Transformation of HSP Enhances Pharmaceutical Performance

HSP is undergoing a solid-state change post encapsulation in SLN is validated by the thermal and crystalline analyses. Pure HSP displayed a sharp melting peak and distinct diffraction pattern due to the crystalline nature of the drug. The sharp melting and distinct diffraction patterns were not observed in the HSP-HA-SLN formulation, suggesting the drug was present in an amorphous or molecularly dispersed form in the lipid matrix. This change to an amorphous state from a crystalline state is an advantage from a formulation perspective. Amorphous drug forms generally show higher apparent solubility and greater thermodynamic activity, which can aid in improved drug bioavailability at the site of application [72]. The lipid matrix, particularly Compritol^®^ 888 ATO / glyceryl behenate, is known to produce an imperfect and polymorphic crystalline structure that can help by accommodating drug molecules within lattice imperfections [73]. Moreover, no exothermic degradation events were detected till the temperature of 300 °C, indicating that the formulation is thermally stable. This stability supports its suitability for further processing, storage, and potential clinical use.

### Controlled Release Behavior Supports Sustained Antioxidant Action

The in vitro release results showed that HSP-HA-SLN released the drug slowly over 24 h, with the absence of any significant burst release during the initial time points. The low release observed during the initial phase is indicative of HSP being retained within the lipid core rather than loosely bound to the particle surface. Release kinetics were best described by the Higuchi model, indicating diffusion-controlled release from a solid matrix. This type of release behaviour is typical for well-formed SLNs and is consistent with drug diffusion through lipid domains that develop as the matrix hydrates and reorganizes in the release medium [74, 75]. The good fit to the Korsmeyer–Peppas model further supports diffusion as the dominant release mechanism, with little contribution from matrix erosion or swelling [76]. For radiation-induced skin injury, a sustained release profile is desirable because oxidative stress and inflammation are not short-lived processes. Maintaining local drug levels over time may help reduce ROS and associated inflammatory responses, while limiting the need for repeated application.

### Cytocompatibility and Concentration-dependent Biological Activity

Cytotoxicity studies in A431 cells showed that the SLN carrier system is biocompatible. Blank SLNs showed that the lipid matrix and surfactants do not cause cellular toxicity by maintaining high cell viability at all tested concentrations. Both free HSP and HSP-HA-SLN were well tolerated at lower concentrations relevant to topical antioxidant therapy, keeping cell viability above 90%. Both free HSP and HSP-HA-SLN showed a concentration-dependent decrease in viability at higher concentrations, which is in line with HSP’s known antiproliferative activity in carcinoma cell lines. Crucially, HSP-HA-SLN did not show disproportionate toxicity in comparison to the free drug, suggesting that the nanocarrier does not introduce extra cytotoxic effects. Instead of carrier-induced toxicity, enhanced cellular uptake mediated by surface functionalization may be responsible for the slightly larger reduction in viability seen for HSP-HA-SLN at higher doses [77]. This dual behavior underscores the formulation’s safety within the intended topical therapeutic window while also highlighting its potential relevance in oncological contexts at elevated concentrations.

### Enhanced Intracellular Antioxidant Efficacy through Nanocarrier-mediated Delivery

The HSP-HA-SLN’s discerning improvement of intracellular antioxidant efficacy is a noteworthy biological result of this investigation. Because PMA triggers downstream NADPH oxidase pathways and pro-kinase C-dependent signaling, which leads to strong intracellular ROS generation, PMA-stimulated PBMCs represent a well-established model of oxidative stress [78]. The oxidative stress model was validated in this study when PMA stimulation resulted in a significant increase in intracellular ROS. Interestingly, intracellular ROS induced by PMA were not considerably reduced by treatment with free hesperidin, and ROS levels remained similar to those observed in PMA-treated cells. Hesperidin’s intrinsic physicochemical limitations, such as its limited cellular permeability and poor aqueous solubility, which prevent it from accessing intracellular sites of oxidative stress, are responsible for this lack of effectiveness. The lack of any carrier-related antioxidant or placebo effect was also confirmed by blank SLNs’ inability to alter ROS levels.

In contrast, HSP delivered using HA-functionalized SLNs led to a clear reduction in intracellular ROS, with levels close to those seen in untreated control cells. This result indicates that formulation-based delivery is necessary for HSP to exert a measurable antioxidant effect inside cells. The SLN system likely improves interaction with the cell surface and supports intracellular delivery of the drug. In addition, HA on the particle surface may increase contact with cell membranes and aid uptake through bioadhesive interactions. Although specific uptake pathways were not examined in this study, the improved intracellular antioxidant effect is in line with previous reports showing enhanced cellular uptake and biological activity for HA-functionalized nanocarriers [79, 80]. Several studies have reported the antioxidant and anti-inflammatory effects of hesperidin. It has been shown to reduce ROS generation and help maintain epithelial barrier function under inflammatory conditions [81]. HSP has also been reported to lower intracellular ROS levels in PBMCs in models of inflammatory disease [82]. Evidence further suggests that formulation strategies can enhance these effects. For example, SLN-loaded HSP reduced oxidative stress and restored antioxidant enzyme levels in a doxorubicin-induced cardiotoxicity model [83], while optimized SLN-HSP formulations showed improved anti-inflammatory activity in vivo [84].

From a therapeutic point of view, intracellular ROS are a key contributor to radiation-induced skin injury, as they drive keratinocyte damage, endothelial dysfunction, and sustained inflammatory responses. The ability of HSP-HA-SLN to reduce intracellular ROS, therefore supports its potential use in limiting oxidative stress–related skin toxicity. The clear difference observed between free hesperidin, blank SLNs, and HSP-HA-SLN indicates that the antioxidant effect depends on formulation-enabled intracellular delivery rather than on the drug or carrier alone.

### Ex Vivo Skin Permeation and Retention Demonstrate Formation of a Dermal Drug Depot

Ex vivo skin permeation studies offer useful information on how surface properties of a formulation affect drug distribution within the skin. Uncoated HSP-SLN showed moderate permeation but also left a relatively large proportion of drug on the skin surface, suggesting limited interaction between the formulation and skin tissue. In contrast, HA-functionalized HSP-HA-SLN showed a more favorable distribution pattern, with higher dermal retention, increased permeation, and a clear reduction in surface residue. The ~ 36% increase in skin retention observed with HSP-HA-SLN suggests the formation of a stable dermal drug depot, which is desirable for the topical treatment of radiation-induced dermatitis. Retention of the drug within the epidermis and dermis supports prolonged local antioxidant activity at the site of injury [85]. The lower amount of unabsorbed drug on the skin surface also indicates improved wetting, hydration, and bioadhesion, likely due to the presence of the HA coating.

Although the permeation into the receptor compartment was higher for HSP-HA-SLN, this does not indicate uncontrolled transdermal transport. Rather, it reflects increased overall penetration of the drug into and across the skin [86]. The synergism of SLN-mediated occlusion and HA-driven hydration and bioadhesion is the likely reason for the observed outcomes. The lipid matrix contributes to skin hydration and temporary barrier relaxation, while HA promotes close contact with hydrated skin layers and extracellular matrix components [87, 88]. These factors together can help stabilize the drug residence within the skin and reduce early disposition of the drug from the application site.

### Overall Implications, Translational Relevance, and Future Perspectives

These results show that HA functionalization transforms the conventional SLNs into an efficient topical formulation capable of addressing the solubility, permeability, and intracellular delivery limitations of HSP. The controlled drug release, enhanced dermal retention, and improved intracellular antioxidant activity of the HSP-HA-SLN nano-formulation are some of the needed formulation characteristics in the management of radiation-induced skin injury, particularly for addressing the persistent oxidative stress and disruption of cellular redox balance.

The physicochemical characterization results, diffusion-controlled drug release profile, satisfactory cytocompatibility, and consistent suppression of intracellular ROS underscore the potential for translating development activities in future. The promising ex vivo skin distribution profile, as exhibited by increased dermal deposition, indicates the ability of the nano-formulation to form a localized dermal drug depot. This is a desirable characteristic for management of dermal disorders, including radiation-induced dermatitis, where sustained local drug presence is preferred, and excess systemic exposure is not desired. To summarize, these results highlight the potential of rational surface functionalization to improve the topical performance of antioxidant formulations for radioprotective applications.

However, the current study focused on performance characterization through in vitro and ex vivo models, which are unable to simulate closely the intricate vascular, inflammatory, and regenerative processes involved in radiation-induced skin injury in vivo. Additional studies utilizing animal models of radiation dermatitis that have been validated will be required to verify therapeutic efficacy, safety, and ideal dosage schedules. Likewise, while HA functionalization greatly improved biological performance, the precise cellular uptake mechanisms were not investigated. Nanocarrier performance may be further improved by clarifying uptake pathways and evaluating the impact of HA molecular weight and surface density. Finally, assessment of long-term stability, repeated topical application, and performance on compromised skin barriers will be important steps toward clinical translation.

## Conclusions

This study describes the development of a HA-functionalized solid lipid nanoparticle system for the topical delivery of HSP, with the aim of addressing key biological and formulation challenges associated with radiation-induced skin injury. The formulation was designed to improve several known limitations of free HSP, including low aqueous solubility, high crystallinity, limited penetration into skin layers, and poor intracellular availability. By combining a lipid-based carrier system with HA surface modification, the approach provides a practical means of improving both drug localization within the skin and cellular access at sites of oxidative stress. A Quali-ty-by-Design-guided optimization strategy enabled controlled adjustment of formulation variables, resulting in nanoparticles with particle sizes below 300 nm and high drug entrapment efficiency. Physicochemical characterization confirmed successful incorporation of HSP in an amorphous form within a stable lipid matrix, along with effective HA surface coating. These properties contributed to good colloidal stability and surface characteristics suitable for topical application. Functionally, the optimized formulation showed sustained, diffusion-controlled drug release, high cytocompatibility, and improved retention within skin tissue.

Importantly, HA-functionalized SLNs produced a marked improvement in intracellular antioxidant activity, as demonstrated by significant suppression of PMA-induced ROS generation in human PBMCs compared with free HSP. This result highlights the role of nanocarrier-based delivery in allowing antioxidant compounds to act effectively at intracellular sites of oxidative stress. Ex vivo skin permeation experiments also showed that HA functionalization improved drug distribution within the skin, with higher dermal retention, lower surface residue, and controlled permeation across the tissue. Such a balance between skin retention and permeation is particularly relevant in radiation-induced skin injury, where sustained local drug action is more desirable than systemic exposure. Collectively, these findings indicate that HA-functionalized SLNs represent a suitable platform for topical antioxidant delivery. By improving formulation stability, localization within the skin, and intracellular drug availability, this system offers a potential practical approach for reducing oxidative stress–related skin toxicity during radiotherapy. While further in vivo and clinical studies are needed to confirm long-term safety and therapeutic benefit, the present results provide a clear basis for continued development of nanocarrier-based treatments for application in radiation-induced skin injury and related inflammatory skin conditions.

## Supplementary Information

Below is the link to the electronic supplementary material.

Supplementary Material 1

## Data Availability

The datasets generated and/or analyzed during the current study are available from the corresponding author on reasonable request.
