# Supplementary material for: Hyaluronic Acid-functionalized Hesperidin-loaded Solid Lipid Nanoparticles for Mitigating Oxidative Stress: A Potential Strategy for Radiation-induced Skin Injury
Source: Appl Biochem Biotechnol. 2026 Apr 28;198(8):5716–43. doi: 10.1007/s12010-026-05714-w (PMC13408121; doi:10.1007/s12010-026-05714-w)
Supplement: Supplementary file 1 — Supplementary Material 1 [file 12010_2026_5714_MOESM1_ESM.docx]

**Supplementary Data**

**FTIR analysis**

Fourier-transform infrared (FTIR) spectroscopy was employed to investigate potential chemical interactions between formulation components, assess drug encapsulation, and confirm surface modification of solid lipid nanoparticles (SLNs) with hyaluronic acid (HA). FTIR spectra of hesperidin (HSP), blank SLNs, and HA-coated HSP-loaded SLNs (HSP-HA-SLN) were recorded using a Bruker FTIR spectrometer (Germany). Samples were analyzed over a wavenumber range of 4000–650 cm⁻¹ at room temperature with appropriate background subtraction. The spectra were compared to identify characteristic functional groups, changes in band intensity, peak broadening, or minor shifts that could indicate physical interactions, encapsulation of HSP within the lipid matrix, and HA adsorption onto the SLN surface.

**Result** **and Interpretation:** The FTIR spectrum of HSP displayed distinct characteristic absorption bands associated with its polyphenolic and flavonoid structure. A broad absorption band observed in the 3200–3600 cm⁻¹ region was attributed to phenolic O–H stretching vibrations, reflecting the presence of multiple hydroxyl groups. Peaks appearing in the 2900–3000 cm⁻¹ region corresponded to aliphatic and aromatic C–H stretching vibrations. The absorption bands detected in the 1600–1750 cm⁻¹ region were assigned to carbonyl (C=O) stretching and aromatic C=C vibrations. Additionally, strong bands in the 1000–1300 cm⁻¹ fingerprint region were associated with C–O and C–O–C stretching modes, consistent with glycosidic and ether functionalities present in HSP [Binkowska 2020; Krysa, Szymańska-Chargot, and Zdunek 2022]. The blank SLN spectrum exhibited characteristic bands related to the lipid components of the nanoparticle matrix. Prominent aliphatic C–H stretching vibrations were observed at approximately 2850–2920 cm⁻¹, along with a strong ester carbonyl stretching band near 1730–1750 cm⁻¹, confirming the presence of esterified lipid excipients. The overall spectral profile of blank SLNs was consistent with a lipid-based nanocarrier system and did not show additional peaks indicative of chemical modification. The FTIR spectrum of HSP-HA-SLN largely retained the characteristic absorption bands of the lipid matrix, indicating preservation of the SLN structural integrity following drug loading and HA coating [Mohseni et al. 2019]. Compared to blank SLNs, the HSP-HA-SLN spectrum showed subtle broadening and increased intensity in the O–H stretching region (3200–3600 cm⁻¹), consistent with the hydroxyl-rich structure of HA and potential hydrogen-bonding interactions at the nanoparticle surface. Minor changes in band intensity within the fingerprint region (1000–1300 cm⁻¹) were also observed, which may reflect the contribution of HA polysaccharide groups and the presence of encapsulated HSP. Importantly, no new absorption bands or pronounced peak shifts were detected, suggesting the absence of chemical incompatibility or covalent bond formation between HSP, HA, and the lipid matrix [Mehmood et al. 2024]. These observations support the physical encapsulation of HSP within the SLNs and the successful surface coating of SLNs with HA through non-covalent interactions, such as hydrogen bonding and electrostatic associations.


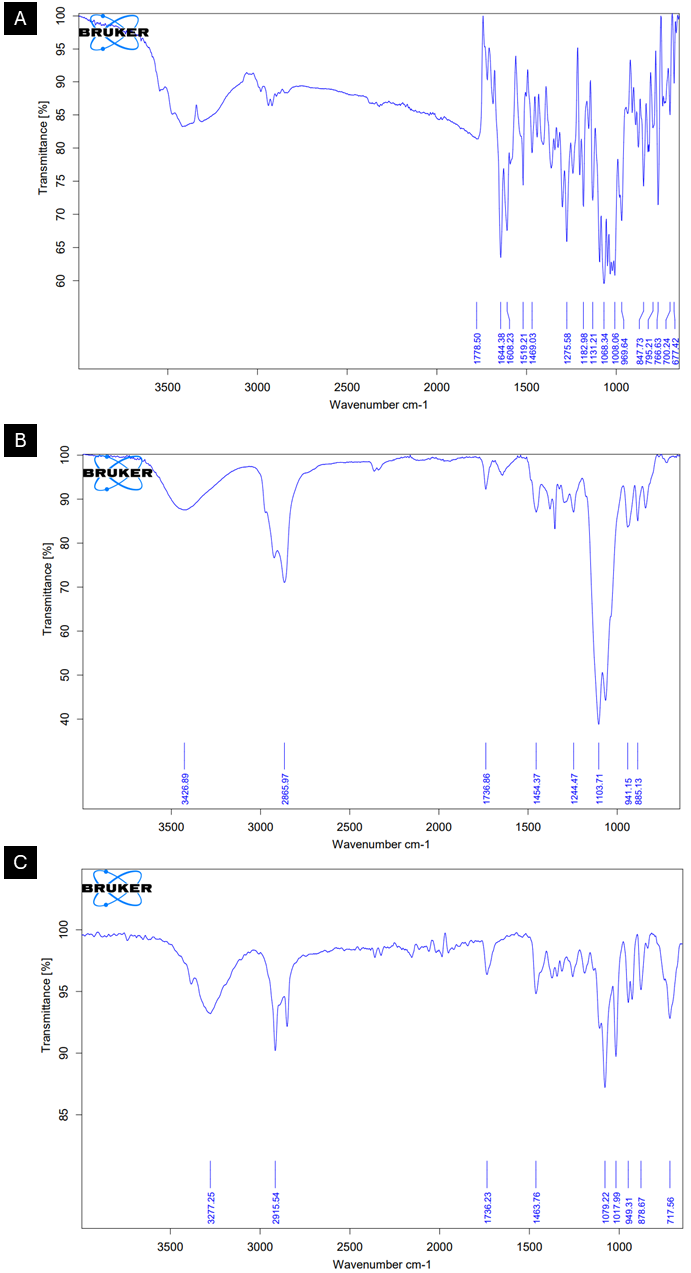


**Figure S1. FTIR spectra of (A) hesperidin (HSP), (B) blank solid lipid nanoparticles (Blank SLN), and (C) hyaluronic acid–coated hesperidin-loaded SLNs (HSP-HA-SLN).** HSP exhibits characteristic phenolic O–H stretching, C–H stretching, carbonyl/aromatic vibrations, and C–O/C–O–C bands. Blank SLNs display lipid-associated aliphatic C–H and ester carbonyl stretching bands. The HSP-HA-SLN spectrum retains the SLN lipid features with subtle broadening in the O–H region and changes in the fingerprint region, consistent with HA surface coating and physical encapsulation of HSP without evidence of chemical incompatibility.

**Ex vivo Drug Permeation and Retention across Full-Thickness Skin**

**Figure S2: Ex vivo cumulative permeation of hesperidin (HSP) from solid lipid nanoparticles (HSP-SLN) and hyaluronic acid-coated SLN (HSP-HA-SLN) across excised skin over 24 h. Data are expressed as mean ± SD (n = 6).** HSP-HA-SLN demonstrated a significantly higher permeation rate and cumulative drug transport, indicating improved skin penetration and sustained release behavior. HSP-HA-SLN demonstrated a significantly higher permeation rate and cumulative drug transport, indicating improved skin penetration and sustained release behavior.

**Supplementary References**

Binkowska, I. 2020. “Hesperidin: Synthesis and Characterization of Bioflavonoid Complex.” SN Applied Sciences. 2(3): 445.

Krysa, M., M. Szymańska-Chargot, and A. Zdunek. 2022. “FT-IR and FT-Raman Fingerprints of Flavonoids – A Review.” Food Chemistry. 393: 133430.

Mehmood, Y., H. Shahid, S.M. Rizvi, U. Jamshaid, N. Arshad, M. Nur-e-Alam, M.D. Hussain, and M. Kazi. 2024. “Hyaluronic Acid-Solid Lipid Nano Transporter Serum Preparation for Enhancing Topical Tretinoin Delivery: Skin Safety Study and Visual Assessment of Skin.” Frontiers in Pharmacology. 15.

Mohseni, R., Z. ArabSadeghabadi, N. Ziamajidi, R. Abbasalipourkabir, and A. RezaeiFarimani. 2019. “Oral Administration of Resveratrol-Loaded Solid Lipid Nanoparticle Improves Insulin Resistance Through Targeting Expression of SNARE Proteins in Adipose and Muscle Tissue in Rats with Type 2 Diabetes.” Nanoscale Research Letters. 14(1): 227.
